# Mining for humoral correlates of HIV control and latent reservoir size

**DOI:** 10.1371/journal.ppat.1008868

**Published:** 2020-10-13

**Authors:** Jishnu Das, Anush Devadhasan, Caitlyn Linde, Tom Broge, Jessica Sassic, Max Mangano, Sean O'Keefe, Todd Suscovich, Hendrik Streeck, Alivelu Irrinki, Chris Pohlmeyer, Gundula Min-Oo, Shu Lin, Joshua A. Weiner, Thomas Cihlar, Margaret E. Ackerman, Boris Julg, Steven Deeks, Douglas A. Lauffenburger, Galit Alter

**Affiliations:** 1 Ragon Institute of MGH, MIT and Harvard, Cambridge, United States of America; 2 Department of Biological Engineering, MIT, Cambridge, United States of America; 3 Institute for HIV Research, Essen, Germany; 4 Gilead Life Sciences, San Francisco, CA, United States of America; 5 Thayer School of Engineering, Dartmouth College, Hanover, United States of America; 6 Department of Medicine, University of California San Francisco, San Francisco, United States of America; Vaccine Research Center, UNITED STATES

## Abstract

While antiretroviral therapy (ART) has effectively revolutionized HIV care, the virus is never fully eliminated. Instead, immune dysfunction, driven by persistent non-specific immune activation, ensues and progressively leads to premature immunologic aging. Current biomarkers monitoring immunologic changes encompass generic inflammatory biomarkers, that may also change with other infections or disease states, precluding the antigen-specific monitoring of HIV-infection associated changes in disease. Given our growing appreciation of the significant changes in qualitative and quantitative properties of disease-specific antibodies in HIV infection, we used a systems approach to explore humoral profiles associated with HIV control. We found that HIV-specific antibody profiles diverge by spontaneous control of HIV, treatment status, viral load and reservoir size. Specifically, HIV-specific antibody profiles representative of changes in viral load were largely quantitative, reflected by differential HIV-specific antibody levels and Fc-receptor binding. Conversely, HIV-specific antibody features that tracked with reservoir size exhibited a combination of quantitative and qualitative changes marked by more distinct subclass selection profiles and unique HIV-specific Fc-glycans. Our analyses suggest that HIV-specific antibody Fc-profiles provide antigen-specific resolution on both cell free and cell-associated viral loads, pointing to potentially novel biomarkers to monitor reservoir activity.

## Author summary

Current combination antiretroviral therapy (ART) regimens have reversed the death sentence once associated with an HIV diagnosis. However, the virus is never fully eliminated. Rather, latently infected cells with integrated virus (latent reservoir) persist and the virus rebounds rapidly upon discontinuation of therapy. Further, even for those on ART, immune dysfunction, driven by persistent non-specific immune activation, ensues and progressively leads to premature immunologic aging. Current biomarkers monitoring these changes are non-specific–they focus on generic inflammatory changes that may also track with other infections or disease states. In this manuscript, we used an unbiased analytical systems approach to identify antigen-specific biomarkers of HIV disease state/treatment status, active viremia and the latent reservoir. By virtue of them being antigen-specific, these are robust context-specific biomarkers of HIV disease progression, viremia and reservoir size. Our framework highlights the strength of using systems approaches in identifying humoral biomarkers, and can be used in other contexts to identify antigen-specific correlates of infectious disease outcome.

## Introduction

With rapid advances in HIV treatment over the last two decades, current combination antiretroviral therapy (ART) regimens have reversed the death sentence once associated with an HIV diagnosis [1]. Yet, despite their effectiveness in reducing viral replication, and associated loss of CD4+ T cells, the virus is never completely eradicated. Rather, latently infected cells with integrated virus persist [2]. The virus rebounds rapidly upon discontinuation of therapy. Along these lines, even on suppressive ART, infection-associated transcription, in the absence of replication, may persist. This marks the presence of a more active viral reservoir deep within reservoir sites, which may contribute to disease progression, premature immune aging, T cell exhaustion, and immune dysfunction [2]. While several inflammatory markers have been identified that track with persisting viral activity or the latent reservoir size itself, a quantifiable antigen-specific biomarker that provides insight on viral activity, deep within tissues, could provide critical insights to prevent the continued morbidity observed with HIV treatment [3].

Due to stochastic sampling biases and the predominant residence of the viral reservoir in tissues, several host inflammatory markers have been noted that shift with viral load [4–6], HIV disease progression [7, 8] and immune dysfunction [2]. Other T cell phenotypes have been noted to track with HIV-associated progression on ART [9], yet these markers do not track with viral replication or reservoir size. Moreover, due to the non-antigen specific nature of these biomarkers, and the fact that these inflammatory markers shift with additional co-morbidities (such as co-infection with HCV or diabetes), uncertainties remain related to the utility of non-specific biomarkers for clinical management.

Accumulating data suggest that changes in the profiles of antibodies that track with inflammation may serve as critical biomarkers of viral load control and even reservoir size. Early studies highlighted the presence of agalactosylated antibodies (a marker that tracks with inflammation) among individuals with HIV viremia [10]. Additionally, similar changes were recently noted in total non-specific antibodies in ART-treated individuals, tracking with antiviral activity [11]. Bulk glycan-lectin interactions in the host have also been linked to reservoir size and HIV reactivation [12]. These antibody alterations, originally observed in the setting of autoimmunity [13], are now known to be selectively elevated in spontaneous HIV controllers [11], potentially attributable to persistence of HIV within germinal centers where B cell activation may persist [14]. Deeper analysis of the functional consequences of these changes pointed to the consistent detection of higher levels of HIV-specific antibody dependent cellular cytotoxicity (ADCC) among spontaneous controllers of HIV (compared to individuals with naturally progressive infection), as well as the generation of antibodies able to leverage Fc effector functions more effectively among this unique population [15–17]. Whether these antibodies contribute directly to the maintenance of the viral reservoir remains unclear, however, these virus-specific antibody changes clearly shift across clinical phenotypes of viral control. Yet, whether antigen-specific antibody profiles can be used as biomarkers of viral control and even of reservoir size remains unclear.

To begin to explore the possibility of identifying antigen-specific antibody changes that track with HIV clinical phenotypes, viral load and the latent reservoir, a systems serology approach [18, 19] was utilized to comprehensively profile HIV-specific biophysical and functional profiles of antibodies in a cohort of 78 subjects including those controlling HIV in absence of therapy (elite and viremic controllers), those on effective ART, and those with untreated chronic infection and higher viral loads. We also included HIV uninfected individuals as controls who should not have any HIV antigen-specific humoral responses. Using an unbiased analytical framework, we found HIV-specific humoral biomarkers that both distinguished clinical phenotypes as well as marked viral load and/or reservoir size.

## Results

### Humoral response profiles can accurately distinguish clinical phenotypes

**Fig 1:**
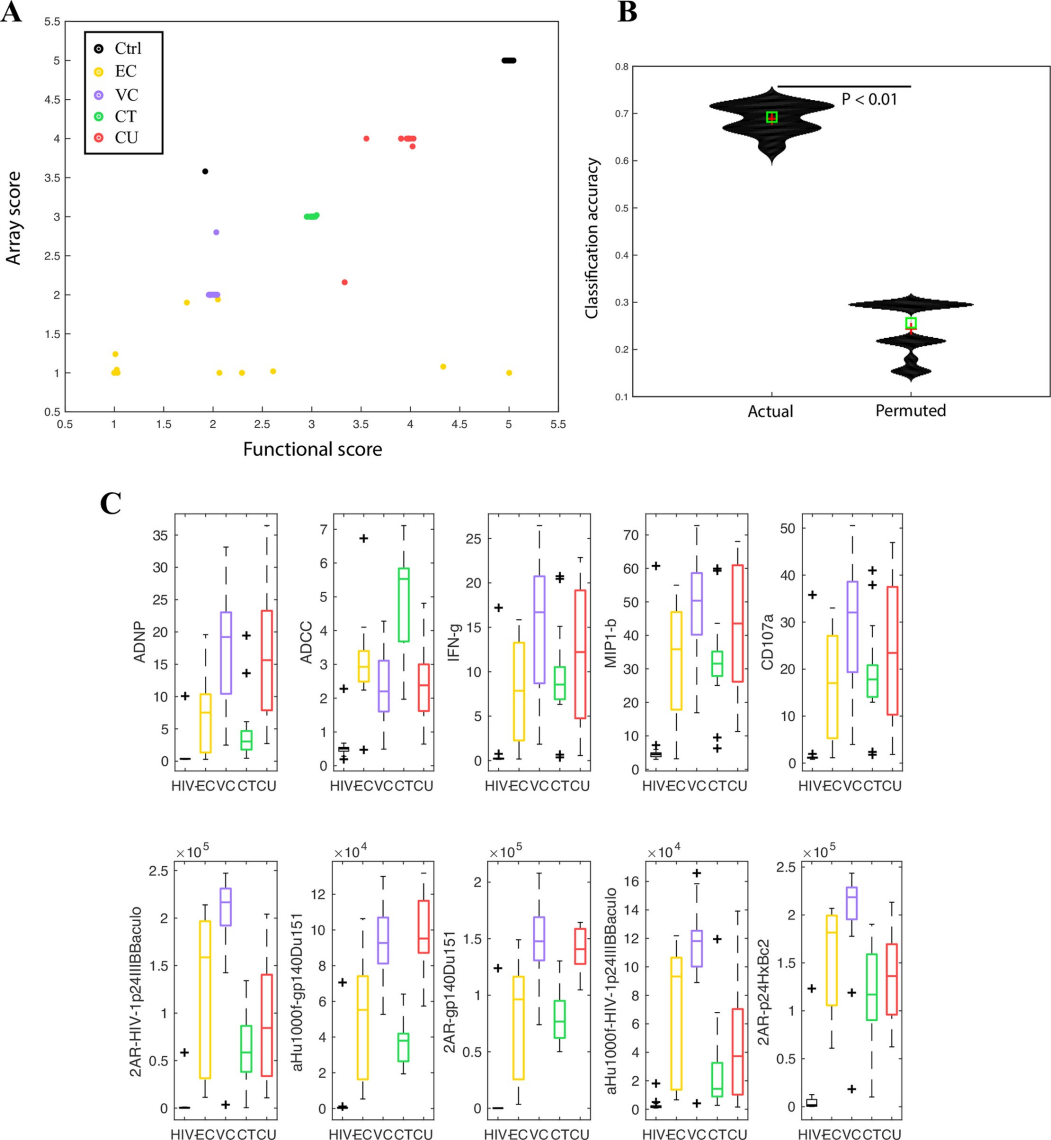
Humoral response profiles can accurately distinguish clinical phenotypes. A. Bi-plot showing how a classifier built on functional (Fc effector function) and array/biophysical data can discriminate between subjects across 5 different HIV clinical phenotypes. The scores on each axis are obtained from a corresponding random forest model. The X axis scores are from a random forest model built on functional data, the Y axis scores are from a random forest model built on array/biophysical data. B. Violin plots showing classification accuracy of the random forest model from 1A on real and permuted data, as measured in a 5-fold cross validation framework (i.e., with data from some subjects blinded/held out as described in the Methods). Exact P value calculated using a permutation test (P < 0.01) confirms significance of model. C. Boxplots illustrating distributions of the humoral responses that are most predictive/discriminative across the 5 clinical phenotypes.

We measured a total of 293 antibody features per plasma sample (S1 Table, S1 Data), including effector functions and biophysical properties, across 78 individuals (S2 Table) comprising 12 subjects who spontaneously control HIV to below detectable viremia levels (< 40 copies/ml) in the absence of ART–termed elite controllers (ECs), 23 subjects who spontaneously control HIV in the absence of ART but have detectable viremia levels (40–2000 copies/ml)–termed viremic controllers (VCs), 17 subjects on ART with undetectable viremia (CTs, < 40 copies/ml), 12 patients not on ART with detectable viremia (CUs, >2000 copies/ml), and 14 HIV uninfected subjects. While previous data suggested that antibody features are differently correlated across different HIV clinical phenotypes [15, 20], here we used a systems approach to determine whether unique humoral profiles exist that could distinguish subjects independent of viral loads and CD4+ T cell counts. Since the number of serological measurements for each subject is greater than the number of subjects (i.e., our data is high-dimensional), we used machine learning methods appropriate for high-dimensional data that avoid overfitting. Specifically, we used a regularized model, as well as a tree-based classification approach to discriminate between the different clinical phenotypes. We found that a random forest classifier that separately incorporates functional and biophysical responses of these subjects achieved the best separation between all five clinical phenotypes (Fig 1A). Accuracy of classification was assessed using a rigorous five-fold cross validation framework. We divided subjects randomly into five subsets. This was done such that for each fold, four subsets served as the training set—i.e., these four subsets of the data are incorporated into the model training process, and the fifth subset served as the test set—i.e., the model training process is blinded to this subset and the performance of the model is tested on this blinded subset. Each subset served as the test set once–so at the end of each cross-validation run, each subject was in the test fold (blinded fold) exactly once. This was repeated for multiple ways in which the data can be split into five folds (see Methods for additional details). Our framework allows for the rigorous evaluation of model performance when some data is blinded/held out. In this rigorous five-fold cross-validation framework, we found that the model had excellent classification accuracy (Fig 1B). Further, this accuracy was significantly higher using real data, as opposed to permuted data (*P* < 0.01), confirming the robustness of the antibody signature (Fig 1B). Thus, despite similar CD4+ T cell counts and viral loads across several of the subject groups (i.e., EC and CT), antibody profiles were sufficient to discriminate between clinical phenotypes. The performance of our classifier remained equally robust and significant when the analyses were performed only with the HIV+ clinical phenotypes i.e., when the HIV-individuals were excluded (S1 Fig, *P* < 0.01 using a permutation test). A random forest based classifier also outperformed other regularized linear models by ~20% performance in classification accuracy (as measure in a 5-fold cross-validation framework), suggesting there are non-linear differences across these clinical phenotypes.

To define a minimal features that contributed to the classifier, the performance of the classifier was evaluated after each feature was dropped from the overall multivariate model, representing a well-accepted approach to measure out-of-bag classification errors [21]. Using this approach, we found that 5 functional and 5 biophysical measurements were most critical for classifier performance. The functional measurements included antibody-dependent phagocytosis mediated by neutrophils, antibody-dependent cellular cytotoxicity (ADCC) by natural killer (NK) cells, and antibody-mediated degranulation by NK cells (as quantified by interferonγ- IFNγ-, MIP1beta—MIP1β secretion and CD107a expression). The biophysical measurements included gp120-specific antibody binding to Fc-γ-receptor 2A. Together, functional and Fc-receptor binding differences, reflecting qualitative changes in antibodies, were associated with group separation.

Interesting patterns emerged with respect to the humoral measurements that drove separation between clinical phenotypes. First, subjects with undetectable viral loads, including ECs and ART treated subjects, had lower neutrophil-mediated phagocytosis and NK degranulation, but higher ADCC responses than subjects with detectable viral loads–VCs and CUs (Fig 1C, *P* < 0.01). These data highlight the presence of distinct functional profiles linked to viremia. Conversely, ECs and VCs both exhibited enhanced binding to FcγR2A, the Fc-receptor involved in driving phagocytosis [22], compared to ART treated subjects (Fig 1E), suggesting that spontaneous control of HIV is associated with the induction of more functional antibodies irrespective of viral levels–for example, increased ADCC has previously been reported in controllers. These results suggest that HIV-specific antibody qualitative features can clearly distinguish between HIV clinical phenotypes and point to specific antibody effector functions that are largely viral load driven, and Fc-receptor binding profiles that are enriched among controllers of HIV (and hence may be novel pathway associated with virus control).

### Humoral profiles that track with viral load control

Given the predictive power of antibody profiles for HIV clinical status, we next aimed to define whether antibody profiles tracked with viremia. Several studies have noted direct relationships between antibody function and viral loads [23, 24], thus we next examined changes in antibody profiles specifically across subjects with undetectable and detectable viral loads: ECs, VCs, and CUs. Two separate analyses were performed aimed at defining: 1) humoral changes across controllers (EC+VC) and non-controllers (CUs), as well as 2) between controllers–comparing ECs and VCs. Given the high dimensionality of the data, antibody features were initially reduced using a LASSO-based feature selection, followed by classification using the LASSO-selected features and visualization using partial least squares discriminant analysis (PLSDA). The analytical framework is similar to what has been previously described–the use of LASSO helps minimize overfitting with high-dimensional data [19].

**Fig 2:**
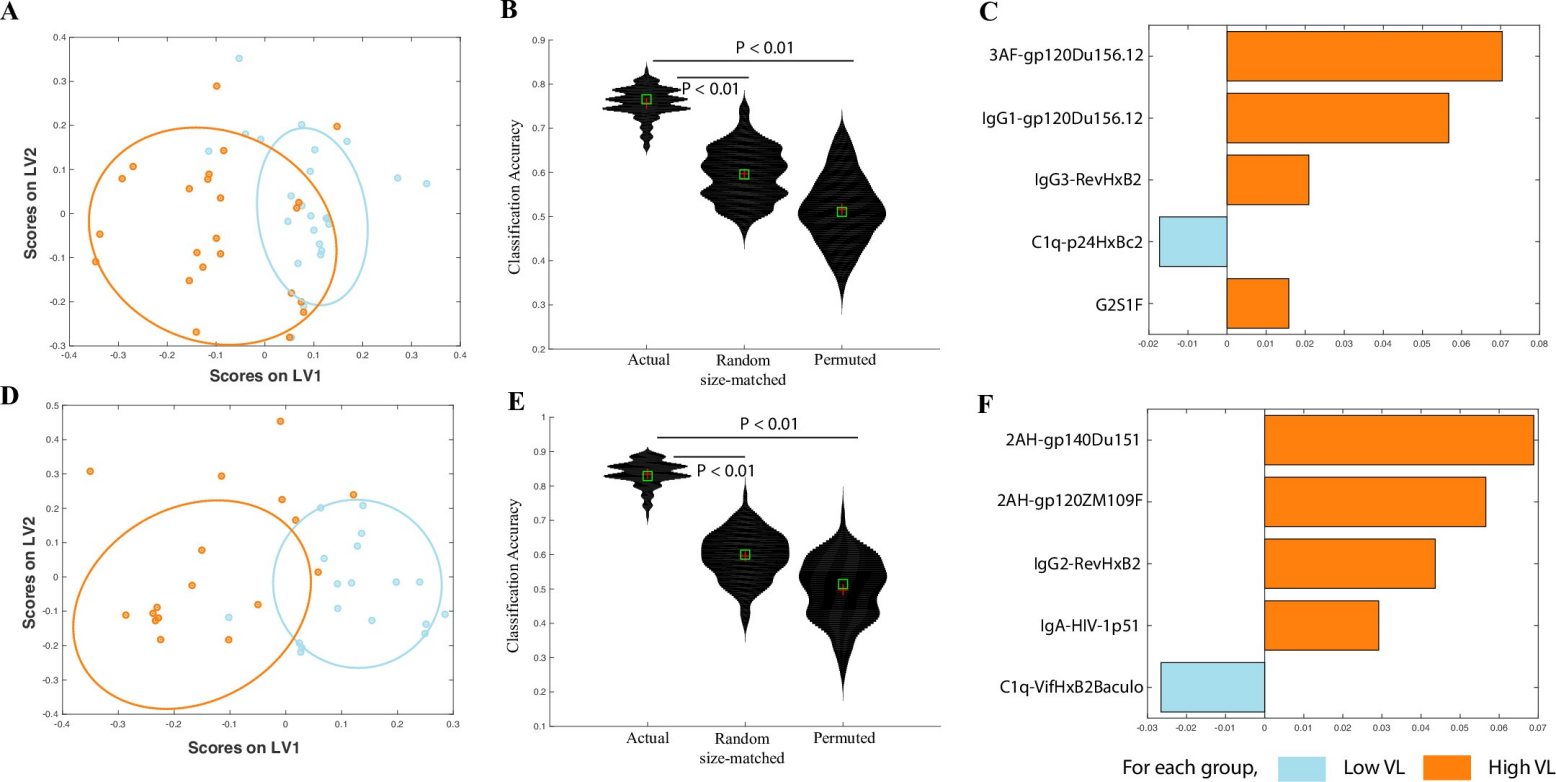
Correlates of viral load in controllers and chronic progressors. A. A LASSO-based model is used to classify controllers and progressors by viral load. The LASSO-selected features are visualized in 2 dimensions using a partial least squares discriminant analysis (PLSDA) latent variable (LV) scores biplot. B. Violin plots showing classification accuracy of the actual model from (a) and of 2 negative control models (based on randomly selected features & permuted data), as measured in a 5-fold cross validation framework. Exact P values (actual vs permuted and actual vs random-size matched) confirm significance of the model. C. PLS variable importance in the projection (VIP) plot corresponding to the features in (a) used to classify subjects by viral load. D. A LASSO-based model is used to classify only controllers by viral load. The LASSO-selected features are visualized in 2 dimensions using a PLSDA LV scores biplot. E. Violin plots showing classification accuracy of the actual model from (d) and of 2 negative control models (based on randomly selected features & permuted data), as measured in a 5-fold cross validation framework. Exact P values (actual vs permuted and actual vs random-size matched) confirm significance of the model. F. PLS VIP plot corresponding to the features in (d) used to classify subjects by viral load.

To be unbiased, we defined whether antibody profiles could simply predict two viral load groups: high and low- based on the median viral load across all subjects (~500 RNA copies/ml). Separation was observed across subjects with low and high viral loads (Fig 2A and 2B). Only 5 of the 293 features were needed to drive the separation between these groups, marked by elevated levels of IgG1 and IgG3 titers, enhanced FcγR3a binding (3AF-gp120Du156.12), and elevated levels of total G2S1F glycans in subjects with higher viral loads (Fig 2C). Conversely, subjects with low or undetectable viral loads uniquely exhibited higher levels of C1q fixing antibodies to Gag p24 (Fig 2C). Overall, these data point to both quantitative differences in antibody titers and associated FcR binding profiles across distinct HIV antigens that track with viral loads. Further, the identified biomarkers of viral load were not significantly associated with a large panel of cytokines, suggesting that non-specific cytokines cannot replace the antigen-specific changes observed in this manuscript (S2 Fig).

We then repeated similar analyses with only the controllers (ECs and VCs) to define whether unique humoral biomarkers could discriminate between controllers with undetectable or detectable viral loads. We sought to determine whether antibody profiles could discriminate between controllers with undetectable or very low viremia (<75 copies/ml) and controllers with detectable viremia (>75 copies/ml). Excellent discrimination was achieved across the HIV-specific antibody profiles across these two groups (Fig 2D and 2E). Again, only five features were required to separate these groups (Fig 2F). Specifically, humoral profiles in the viremic individuals were marked by higher FcγR2A binding (2AH-gp140Du151 and 2AH-gp120ZM109F), higher IgG2 titers to Rev (with IgG2 being a less functional subclass), and elevated IgA titers to Reverse Transcriptase (p51) (Fig 2F). These data also point to a link between viral load and quantitative differences in antibody titers and associated FcR binding profiles.

### Humoral biomarkers of reservoir size in controllers and chronic progressors

Beyond humoral biomarkers of active viral replication that track strongly with viral burden, significant differences have been noted in the size of the HIV latent viral reservoir among spontaneous controllers of HIV. Thus we next sought to explore whether HIV-specific antibody profiles could also resolve differences in latent reservoir size. For these studies we used quantitative assessments of the total HIV DNA as a measure of the reservoir; although the majority of HIV DNA is defective, it has been shown that total DNA correlates with more precise measures of the reservoir such as IPDA and VOA [25]. Again, two separate analyses were performed: 1) comparing across all individuals off ART, and 2) across individuals on ART.

**Fig 3:**
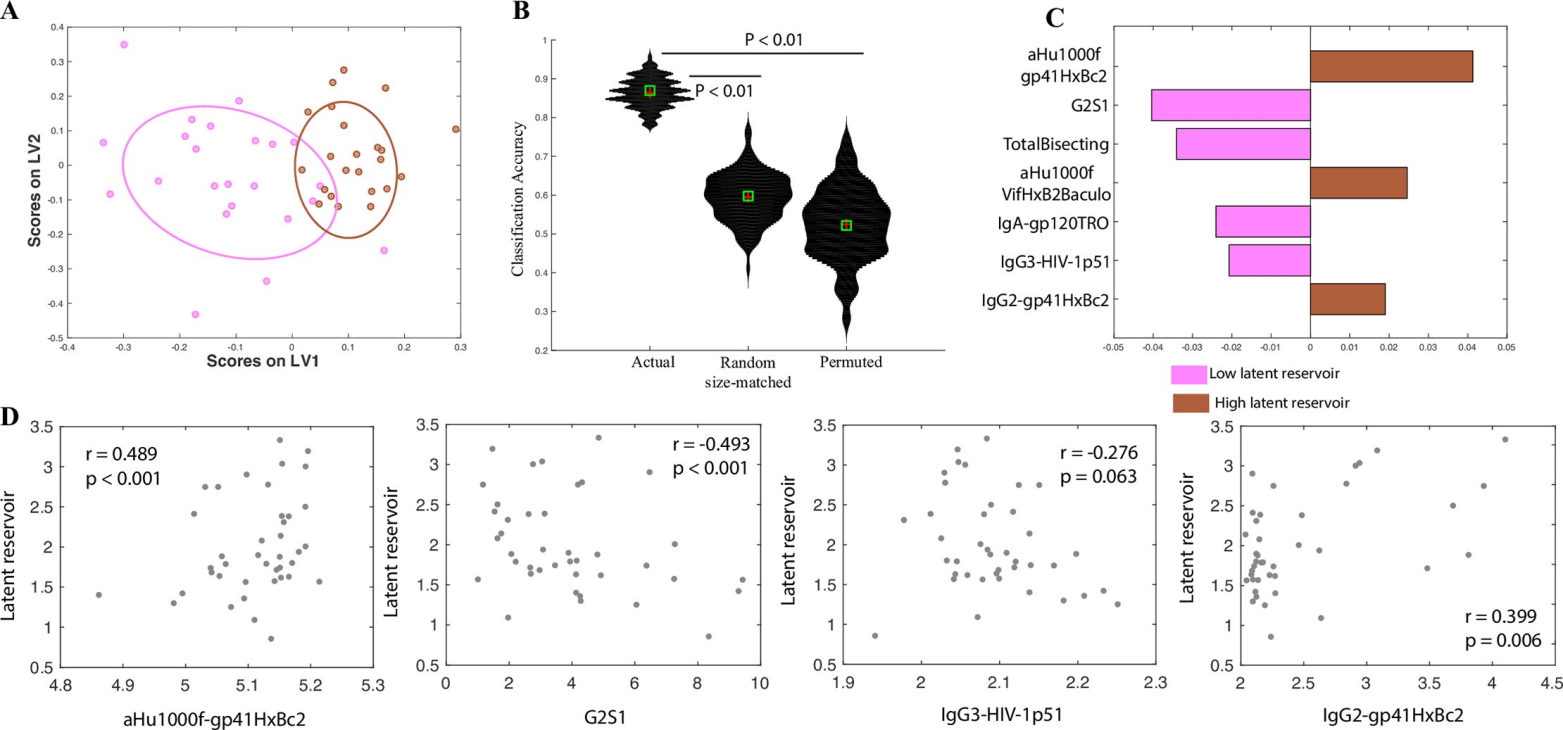
Correlates of latent reservoir size in controllers and chronic progressors. A. A LASSO-based model is used to classify controllers and progressors by latent reservoir size. The LASSO-selected features are visualized in 2 dimensions using a PLSDA LV scores biplot. B. Violin plots showing classification accuracy of the actual model from (a) and of 2 negative control models (based on randomly selected features & permuted data), as measured in a 5-fold cross validation framework. Exact P values (actual vs permuted and actual vs random-size matched) confirm significance of the model. C. PLS VIP plot corresponding to the features in (a) used to classify subjects by latent reservoir size. D. Correlations between biomarkers of the latent reservoir in controllers and progressors, and actual latent reservoir size.

We first sought to define whether antibody profiles tracked with the estimated reservoir size across ECs, VCs and CUs. Using a median split on the distribution of HIV DNA levels across the subjects, LASSO-based feature selection was performed, followed by classification using the LASSO-selected features and visualization using PLSDA. HIV-specific antibody profiles were again able to discriminate between individuals with higher and lower HIV DNA levels (Fig 3A and 3B). The subjects with higher HIV DNA levels had higher Vif-specific IgG titers and higher gp41-specific IgG and IgG2 titers (Fig 3C). However, those with lower HIV DNA levels had functional antibody profiles marked by elevated gp120-specific IgA and Reverse Transcriptase IgG3 titers (Fig 3C). Further, these subjects also had higher levels of G2S1 and bisecting glycans on gp120-specific IgGs (Fig 3C). These results suggest that unlike biomarkers that track with viral load control, biomarkers that track with the estimated size of the latent reservoir size target a range of gene products and are not only associated with quantitative changes in antibody profiles, but also mark qualitative changes in the HIV-specific antibody profiles overall. To further validate that the identified biomarkers aren’t merely tracking with viremia, we sought to identify humoral correlates of latent reservoir size only in controllers (i.e., excluding the chronic untreated group). This eliminates the potential confounding effects of treatment. Again, we were able to identify robust biomarkers that stratify these controllers by reservoir size (S3 Fig, *P* < 0.01 using a permutation test), confirming that our models are able to capture biomarkers specific to the latent reservoir (i.e., they are not generic biomarkers of viremia).

We also sought to explore whether the observed correlates track simply with a high/low stratification of the latent reservoir size as determined by HIV DNA levels, or whether they provide a finer resolution of the actual reservoir size. To address this, we first examined correlations between biomarkers from our model with the most significant variable importance in the projection (VIP) scores (Fig 3C), and the estimated latent reservoir size (measured as a continuous variable). We found that both markers: gp41-specific IgG antibodies and G2S1 glycans were significantly correlated (P < 0.001) with the estimated latent reservoir size (Fig 3D). To further evaluate whether only the markers with the most significant VIP scores track with estimated reservoir size or whether other markers also mark the latent reservoir, we computed correlations between p51- and gp41- specific IgG3 and IgG2 antibodies (the two biomarkers with lower VIP scores) and the latent reservoir size. IgG2 antibodies were significantly correlated (P = 0.006), and IgG3 antibodies had a nominal correlation (P = 0.063) with latent reservoir size (Fig 3D). These results demonstrate that the correlates identified track closely with the estimated reservoir size (as measured by HIV DNA), and provide resolution across the spectrum of latent reservoir sizes in these subjects. Further, the identified biomarkers of reservoir size were not significantly associated with a large panel of cytokines, suggesting that non-specific cytokines cannot replace the antigen-specific changes observed in this manuscript (S2 Fig).

### Humoral biomarkers of reservoir size in ART-treated subjects

**Fig 4:**
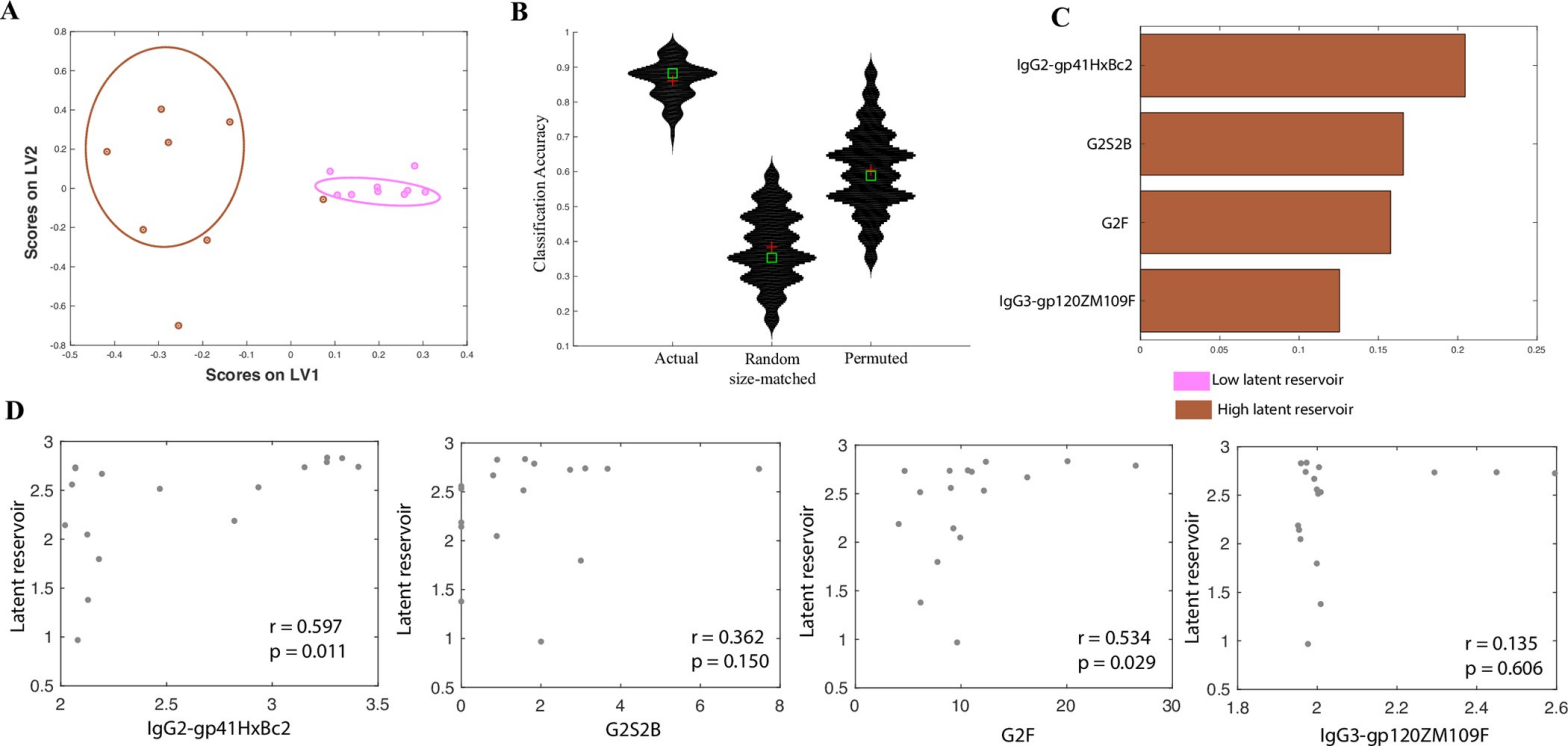
Correlates of latent reservoir size in ART-treated subjects. A. A LASSO-based model is used to classify ART-treated subjects by latent reservoir size. The LASSO-selected features are visualized in 2 dimensions using a PLSDA LV scores biplot. B iolin plots showing classification accuracy of the actual model from (a) and of 2 negative control models (based on randomly selected features & permuted data), as measured in a 5-fold cross validation framework. Exact P values (actual vs permuted and actual vs random-size matched) confirm significance of the model. C. PLS VIP plot corresponding to the features in (a) used to classify subjects by latent reservoir size. D. Correlations between biomarkers of the latent reservoir in ART-treated subjects, and actual latent reservoir size.

Given the emerging need for simple HIV-specific biomarkers to track reservoir changes in ART treated individuals undergoing diverse interventions, a similar analysis was performed to define whether biomarkers could be defined that may be associated with estimated reservoir size in the ART-treated subjects. Excellent separation was achieved between subjects with high and low HIV DNA levels (here, high or low levels was defined using a median split on the distribution of reservoir sizes for only these ART-treated subjects), using HIV-specific antibody profiles alone (Fig 4A and 4B). Only four features were able to separate the groups (Fig 4C). Specifically, the correlates of the size of the estimated latent reservoir size within ART-treated subjects included both gp41- and gp120-specific IgG3 and IgG2 antibodies as well as elevated G2S2B and G2F glycans (Fig 4C). These analyses suggest that antibody profiles that distinguish ART-treated subjects based on latent reservoir size incorporate primarily characteristics driven by subclass profiles. This is in contrast to the profiles that stratify controllers and progressors by latent reservoir size, where a combination of quantitative and functional antibody features provided separation (Fig 3C). However, the quantitative features that provided stratification were similar between both the controllers and progressors group and the ART-treated group. For example, the exact same feature–gp41-specific IgG2 antibodies were a biomarker for higher latent reservoir size in both controllers and progressors (Fig 3C) and the ART-treated group (Fig 4C).

Finally, for these ART-treated subjects, we explored whether the observed correlates track simply with a high/low stratification of the latent reservoir size, or whether they provide a finer resolution of the actual reservoir size. We found that of the 4 biomarkers identified by the model (Fig 4C), 2 markers–gp41-specific IgG2 antibodies and G2F glycans are significantly correlated (P = 0.011 and 0.029 respectively), and a third marker–gp120-specific IgG3 antibodies is nominally correlated (P = 0.15) with latent reservoir size (quantified as a continuous variable). These results demonstrate that these simple antibody features provide meaningful resolution across the spectrum of latent reservoir sizes.

## Discussion

Mounting data suggests that HIV-specific antibodies may differ across HIV-infected patient populations [20], changing both with respect to titers, quality, and functional profiles across clinical phenotypes [15, 20]. Given the antigen-specific nature of these biomarkers, the identification of a precise set of antigen-specific changes may provide a unique opportunity to monitor changes in active viral replication and, more interestingly, in the viral reservoir. Strikingly, using a systems approach, a minimal set of humoral biomarkers were identified able to discretely resolve HIV clinical groups (EC, VC, ART-treated, and CU) as well as act as sensitive measures of changes in reservoir size.

As expected, antibody profiles that tracked with changes in viral replication were associated with both quantitative and qualitative changes in antibodies. Emerging data highlight significant changes in B cell populations and activation in the setting of active viral replication [26, 27]. Robust B cell activation and corresponding responses have been noted in HIV-infected individuals [28], including controllers [29]. Specifically, in HIV infection, plasmablasts arise early and are maintained at high levels in viremic individuals [29]. These changes in B cells are accompanied by hypergammaglobinemia [30, 31], and are consistent with our observations regarding quantitative differences in antibody profiles tracking with active viral replication. However, emerging data suggest that in addition to quantitative changes, alterations in inflammation also program B cells to generate different qualities of antibodies [32]. Thus, qualitative changes in antibody profiles may be a reflection of inflammatory signals from newly activated cells triggered by recent levels of viremia. Importantly, beyond non-specific alterations in antibody properties that track with viral replication, our study elucidates antigen-specific humoral biomarkers that track directly with viral load. Further, while previous studies highlighted distinct coordination profiles among HIV-specific humoral immune profiles within different HIV-infected subject groups [11, 15, 20], here we have identified a small number of humoral features that provide discrete resolution of patient populations using an unbiased systems serology profiling approach.

While viral load quantitation assays provide a remarkably robust approach to monitor viral replicative activity, assays to measure the latent reservoir size and activity in its entirety have been elusive. Several assays have been described that track with the number of latently infected cells or cells that harbor virus that can be reactivated [33]. However, all of these approaches only sample a small proportion of immune cells in an aliquot of blood, which may poorly represent the actual distribution of the virus spatially distributed across tissues and organs. Thus the identified humoral biomarkers may offer a more holistic overview of reservoir size activity, even deep within tissues, providing critical insights into the overall latent reservoir.

While several reactivation assays are in development, here we used viral DNA as a marker of the overall size of both the reservoir that can be reactivated and the defective reservoir [34, 35], due to its reproducible association with time to rebound following treatment interruption [36, 37]. We and others have argued that alterations in HIV-specific antibody profiles may provide a more sensitive measurement of viral reactivation with in tissues [15, 38]. Moreover, because the viral reservoir likely exists in germinal centers, where it may evade the cytotoxic activity of both CD8+ T cells and NK cells, B cells may be able to detect even small changes in reservoir activity, convert to plasmablasts, and then rapidly secrete antibodies to populate the peripheral blood [27]. Thus, unlike sampling biases associated with viral quantification in blood samples, changes in HIV-specific antibodies may provide a picture of activity throughout the body and tissues.

Among the HIV specificities that are linked to reservoir size, specificities beyond those targeting the HIV gp120 envelope protein, the dominant target of functional antibodies, were identified. Specifically, changes in the accessory gene Vif were identified, pointing to the potential induction of antibodies to a broader array of viral targets, potentially from unspliced RNAs, in the setting of reservoir activity. Additionally, gp41-, rather than gp120-specific antibody profiles were linked to reservoir size, highlighting the potential importance of immunodominant regions of the viral envelope, that are often retained on the infected cell surface [39]. Moreover, subjects with higher latent reservoirs have increased binding to Fc receptors and less functional subclasses, while those with lower latent reservoirs have more functional subclasses and glycans. These data potentially suggest that the production of particular subclasses and isotypes, able to mediate specific functions, may contribute to lower latent reservoir size. These data are consistent with previous studies highlighting the presence of elevated levels of functional IgG3 in HIV controllers [40, 41]. Additionally, digalactosylated monosialylated glycoforms were also associated with lower reservoir sizes in controllers and progressors. Galactosylation has been previously tied to enhanced antibody function (primarily ADCC) and sialylation has been tied to reduced inflammation [42]. Our results suggest that antibodies with these specific properties may play a role in helping to maintain a lower reservoir in controllers and progressors. Further, our population was collected to omit changes due to age/sex at the time of recruitment. While, it is possible that changes in antibodies will track with age/sex, this study was not powered to capture these differences as large sample sizes are necessary to observe changes in subclass and glycosylation profiles of antibodies [43]. Rather, we demonstrate that the identified antigen-specific humoral biomarkers accurately capture differences across the spectrums of viral load and latent reservoir size, independent of /distinct from the effect of these clinical parameters.

The markers reported here are a first comprehensive characterization of antigen-specific antibody profiles that mark HIV disease state, viremia, as well as the latent reservoir. Our framework highlights the strength of using systems approaches in identifying humoral antigen-specific biomarkers from serum samples collected at a single time-point. A similar analytical approach can be leveraged in the future to identify and refine biomarkers that also shift longitudinally. While we demonstrate the validity of the identified humoral biomarkers using cross-validation, further validation studies will be required to ultimately define their global clinical value across clades. However, it may be possible to identify antigen-specificities with greater cross-population predictive value using comprehensive profiling with VirScan [44] coupled to systems serology. Because HIV-specific antibody profiling tools may be easily adapted as point of care diagnostics, the development of these tools may represent simple biomarkers for the rapid evaluation of changes in viral replication outside of the blood and changes in the reservoir size.

## Methods

### Ethics

All subjects were recruited from the UCSF-based SCOPE cohort. The UCSF and Massachusetts General Hospital Institutional Review Boards approved the study, and each subject provided written informed consent for participation in the study.

### Subjects

Serum samples from a total of 78 human subjects, including 1) 12 subjects who spontaneously control HIV to below detectable viremia levels (< 40 copies/ml) in the absence of ART–elite controllers (ECs), 2) 23 subjects who spontaneously control HIV in the absence of ART but have detectable viremia levels (40–2000 copies/ml)–viremic controllers (VCs), 3) 17 subjects on ART with undetectable viremia (CTs, < 40 copies/ml), 4) 12 untreated chronically infected progressive patients not on ART (CUs), and 5) 14 HIV- subjects, were used for antibody profiling using our Systems Serology platform. The sample sizes were defined at the start of the study aimed at powering the analysis based on previously quantified associations between antibody measurements and clinical phenotype [15]. There was an approximately equal distribution across the 5 clinical phenotypes.

### Functional and biophysical assays

The functional and biophysical properties of a polyclonal pool of antibodies in the sera of the 78 subjects were profiled using our previously-described systems serology platform [18, 19] (S1 and S2 Tables).

To measure monocyte phagocytosis, a human monocyte cell line (THP-1) -based assay was performed as previously described [45]. Briefly, antigen-coupled green fluorescent beads were incubated with serum for 2 hours at 37°C to allow the formation of immune complexes (ICs). These were then incubated with THP-1 cells for 16 hours, which were then fixed and analyzed by flow cytometry. The percentage of FITC+ THP-1 cells was multiplied by the geometric mean fluorescence intensity of FITC in FITC+ THP-1 cells to determine the antibody dependent cellular phagocytosis (ADCP) score.

To measure neutrophil phagocytosis, antigen-coupled green fluorescent beads were incubated with serum for 2 hours to allow IC formation. These were then incubated with primary leukocytes isolated from fresh whole blood (collected in anticoagulant citrate dextrose tubes) and incubated for 1 hour at 37°C. Gates were drawn on SSChigh CD66b+ CD14- cells, and phagocytic scores were calculated as above.

Antibody-dependent cellular cytotoxicity (ADCC) was assayed using a modified rapid fluorescent ADCC (RFADCC), as previously described [46]. In brief, immunoselected natural-killing resistant human T-lymphoblastoid (CEM-NKr) cells were pulsed with gp120 proteins and labeled with the intracellular dye CFSE and the membrane dye PKH26. Purified IgG (from serum) was added to the labeled, antigen-pulsed CEM-NKr cells after which fresh NK cells were added. The cells were incubated for 4 hours at 37°C and then fixed. The proportion of cells that maintained membrane expression of PKH26 but lost CFSE staining (i.e., lysed cells) were quantified via flow cytometry.

Antibody-dependent complement deposition (ADCD) was assessed via the measurement of complement component C3b deposition on the surface of target cells. Antigen-coupled red fluorescent beads were incubated with serum for 2 hours to allow IC formation. These were then incubated with guinea pig complement for 20 minutes at 37°C, and analyzed by flow cytometry. Complement deposition was reported as median fluorescence intensity on the FITC channel, after gating on singlet, red fluorescent particles.

Ab-dependent NK cell degranulation and cytokine/chemokine secretion were measured as previously described [47]. Antigen-coupled beads were incubated with serum for 2 hours to allow IC formation. These were then incubated with NK cells purified from buffy coats from healthy donors. Gates were drawn on singlet, CD56+/CD3- cells and the results were reported as the percentage of NK cells that expressed surface CD107a, intracellular MIP-1β, or intracellular IFN-γ.

The isotype, subclass, and FcR binding profiles of antigen-specific antibodies were defined using the previously reported multiplexed Fc Array assay [48, 49]. Briefly, uniquely fluorescently coded microspheres were conjugated with HIV antigens of interest, incubated in sera samples, and characterized using a panel of distinct detection reagents. Profiles were quantified in terms of median fluorescent intensities for each measured antigen-specificity and detection reagent pair.

The Fc glycans on antigen-specific IgG were analyzed using a previously described method [50, 51]. Serum samples and gp120-coupled beads were incubated for 1 hour at 37°C with rotation. The coupled beads were then incubated with IdeZ enzyme to cleave the Fc fragments from the antibody-bound beads. These Fc fragments were then treated with PNGase F to remove the glycans from the Fc fragments. These glycans which were then isolated and labeled with APTS dye using a GlycanAssure kit. APTS-labeled glycan samples were analyzed by capillary electrophoresis.

### Classification model for discriminating between clinical phenotypes

We tried different classification models to distinguish subjects across the 5 clinical phenotypes–elite and viremic controllers, subjects on combination antiretroviral therapy (ART), chronic progressors not on ART and healthy HIV- controls. The inputs were the measured humoral immune responses and the model attempted to predict a categorical label corresponding to each clinical phenotype. We attempted to build 1) a regularized model similar to one described previously [19]–a combination of the least absolute shrinkage and selection operator (LASSO)[52] for feature selection and then classification using the LASSO-selected features and 2) random-forest based models using the measured functional and biophysical responses.

The robustness of the model was evaluated using five-fold cross validation replicates. For each five-fold cross validation run, subjects were randomly divided into five subsets. This was done such that for each fold, four subsets served as the training set, and the fifth one served as the test set. Each subset served as the test set once. Thus, for each cross-validation run, each subject was in the test fold exactly once. Thus at the end of each cross-validation run, we obtain a set of predicted arm labels for each subject. We performed 100 independent five-fold cross-validation replicates to account for different ways in which the training and test folds can be split.

The statistical significance of model performance was measured using a “negative control” based on permutation testing [53], by randomly shuffling the data with respect to the arm labels, within a cross validation framework. The features themselves were not shuffled, preserving the correlation structure of the data. These permutations were repeated 100 times to generate a distribution of model accuracies observed in the context of permuted data and randomly selected size-matched feature sets. The entire procedure was repeated across all 5 cross-validation folds. After running through all 5 folds, we compared the predicted label for each subject to the true labels, and obtained a true classification accuracy (equivalent to average classification accuracy across the folds, as the folds are of equal size). We computed the *P* value as the tail probability of the true classification accuracy in the distribution of control model classification accuracies. We reported median *P* values across the independent cross validation replicates. This is an exact P value from a permutation test (multiple hypotheses have not been tested here, and multiple testing correction is not appropriate).

Using this framework, we found that a composite random forest classifier was the most accurate at simultaneously discriminating between the clinical phenotypes. The composite classifier was constructed using a linear combination of two different random forest models–one on the functional measurements, and the other on the biophysical measurements.

### Models for discriminating between subjects based on viral load and latent reservoir size

To discriminate between subjects based on viral load and latent reservoir size, we divided subjects into high/low categories based on a median split. We then used a regularized model similar to one described previously [19] that performs LASSO-based feature selection and classification using the LASSO-selected features. The performance of the model was evaluated in a 5-fold cross-validation framework as described above. For the negative control models, in addition to permutation testing, a second negative control model based on random selection of features, within a 5-fold cross-validation framework was also used. Here, we report an exact P value from a permutation test (multiple hypotheses have not been tested here, and multiple testing correction is not appropriate).

## Supporting information

S1 Fig Humoral response profiles can accurately distinguish clinical phenotypes even within the HIV+ subjects. A. Bi-plot showing how a classifier built on functional (Fc effector function) and array/biophysical data can discriminate between subjects across the 4 clinical phenotypes comprising only the HIV+ subjects. The scores on each axis are obtained from a corresponding random forest model. The X axis scores are from a random forest model built on functional data, the Y axis scores are from a random forest model built on array/biophysical data. B. Violin plots showing classification accuracy of the random forest model from 1A on real and permuted data, as measured in a 5-fold cross validation framework (i.e., with data from some subjects blinded/held out as described in the Methods). Exact P value calculated using a permutation test (P < 0.01) confirms significance of model.
(TIF)

S2 Fig Correlations between markers for viral load/latent reservoir size and cytokines. A. Correlations between 1. antigen-specific humoral markers of viral load for controllers and progressors and 2. a panel of non-specific cytokines. B. Correlations between 1. antigen-specific humoral markers of latent reservoir size for controllers and progressors and 2. a panel of non-specific cytokines.
(TIF)

S3 Fig Correlates of latent reservoir size in only controllers. A. A LASSO-based model is used to classify only controllers by latent reservoir size. The LASSO-selected features are visualized in 2 dimensions using a PLSDA LV scores biplot. B. Violin plots showing classification accuracy of the actual model from (a) and of 2 negative control models (based on randomly selected features & permuted data), as measured in a 5-fold cross validation framework. Exact P values (actual vs permuted and actual vs random-size matched) confirm significance of the model. C. PLS VIP plot corresponding to the features in (a) used to classify subjects by latent reservoir size.
(TIF)

S1 Table List of humoral responses measured in the study. (XLSX)

S2 Table Clinical cohort description (includes clinical category, viral load, CD4 T cell count and estimated reservoir size for each subject). (XLSX)

S1 Data Humoral measurements, viral loads and latent reservoir sizes for subjects in this study. (ZIP)
