# Supplementary figures and images for: Mining for humoral correlates of HIV control and latent reservoir size
Source: PLoS Pathog. 2020 Oct 13;16(10):e1008868. doi: 10.1371/journal.ppat.1008868 (PMC7553335; doi:10.1371/journal.ppat.1008868)

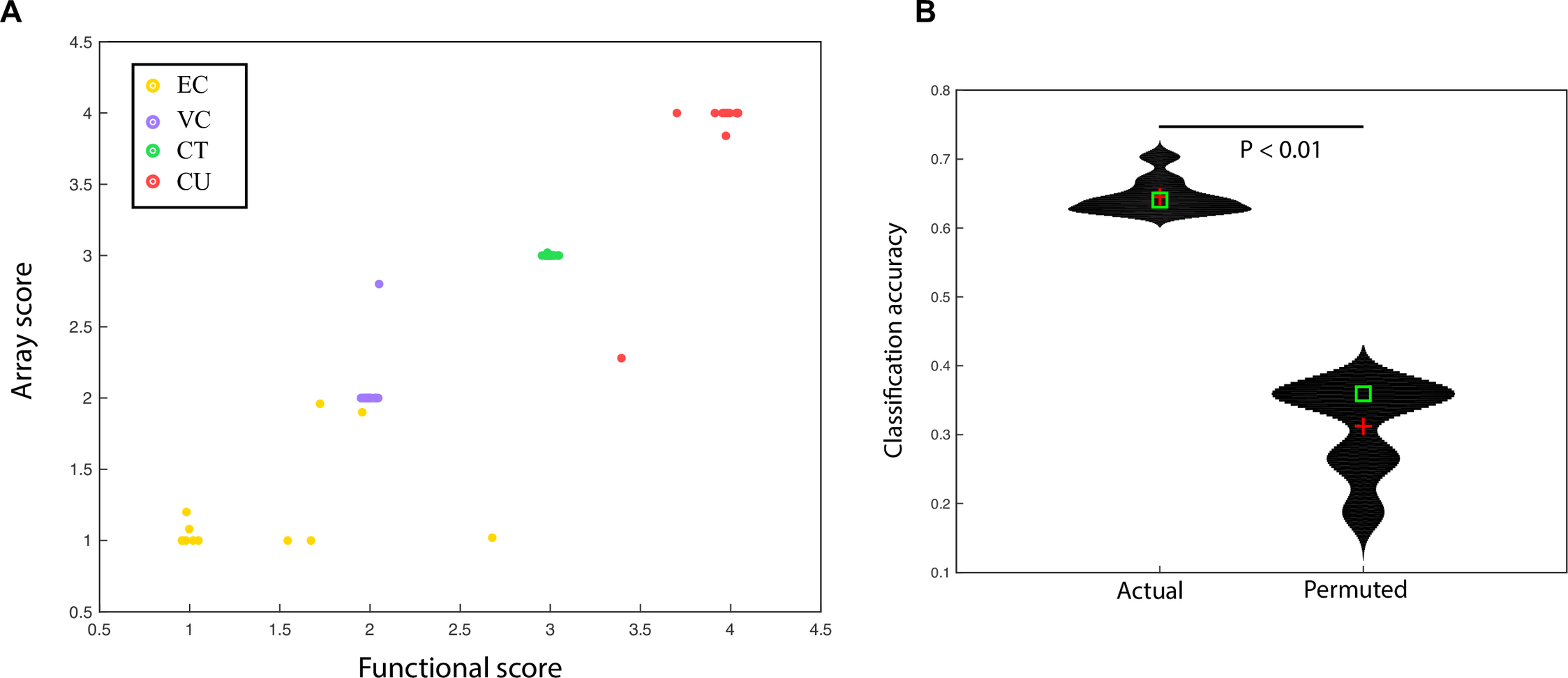

Supplement: S1 Fig — A. Bi-plot showing how a classifier built on functional (Fc effector function) and array/biophysical data can discriminate between subjects across the 4 clinical phenotypes comprising only the HIV+ subjects. The scores on each axis are obtained from a corresponding random forest model. The X axis scores are from a random forest model built on functional data, the Y axis scores are from a random forest model built on array/biophysical data. B. Violin plots showing classification accuracy of the random forest model from 1A on real and permuted data, as measured in a 5-fold cross validation framework (i.e., with data from some subjects blinded/held out as described in the Methods). Exact P value calculated using a permutation test (P < 0.01) confirms significance of model. (TIF) [file ppat.1008868.s001.tif]

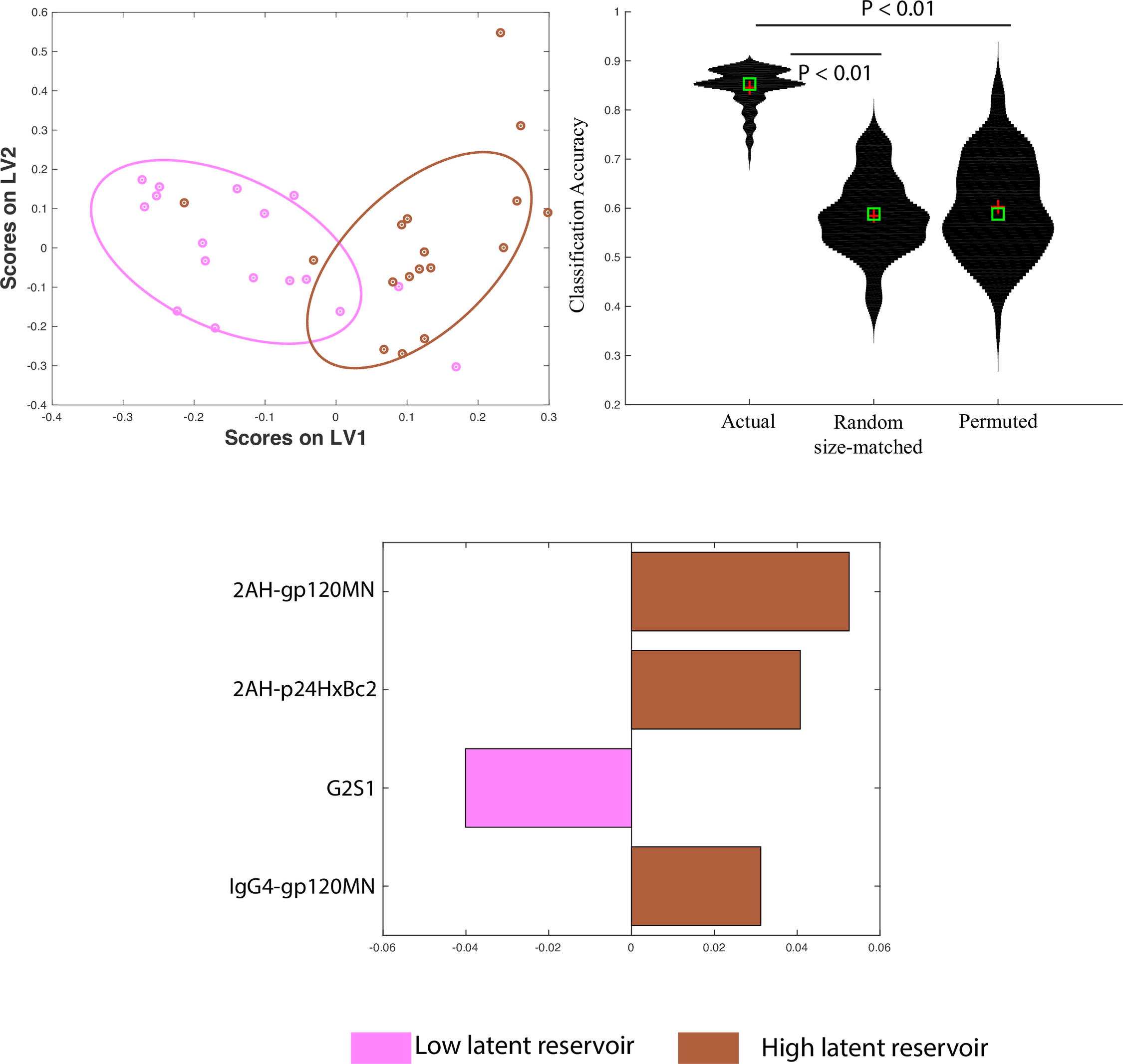

Supplement: S3 Fig — A. A LASSO-based model is used to classify only controllers by latent reservoir size. The LASSO-selected features are visualized in 2 dimensions using a PLSDA LV scores biplot. B. Violin plots showing classification accuracy of the actual model from (a) and of 2 negative control models (based on randomly selected features & permuted data), as measured in a 5-fold cross validation framework. Exact P values (actual vs permuted and actual vs random-size matched) confirm significance of the model. C. PLS VIP plot corresponding to the features in (a) used to classify subjects by latent reservoir size. (TIF) [file ppat.1008868.s003.tif]
